# Supplementary figures and images for: Genome-Wide Investigation of the NAC Transcription Factor Family in Apocynum venetum Revealed Their Synergistic Roles in Abiotic Stress Response and Trehalose Metabolism
Source: Int J Mol Sci. 2023 Feb 26;24(5):4578. doi: 10.3390/ijms24054578 (PMC10003206; doi:10.3390/ijms24054578)

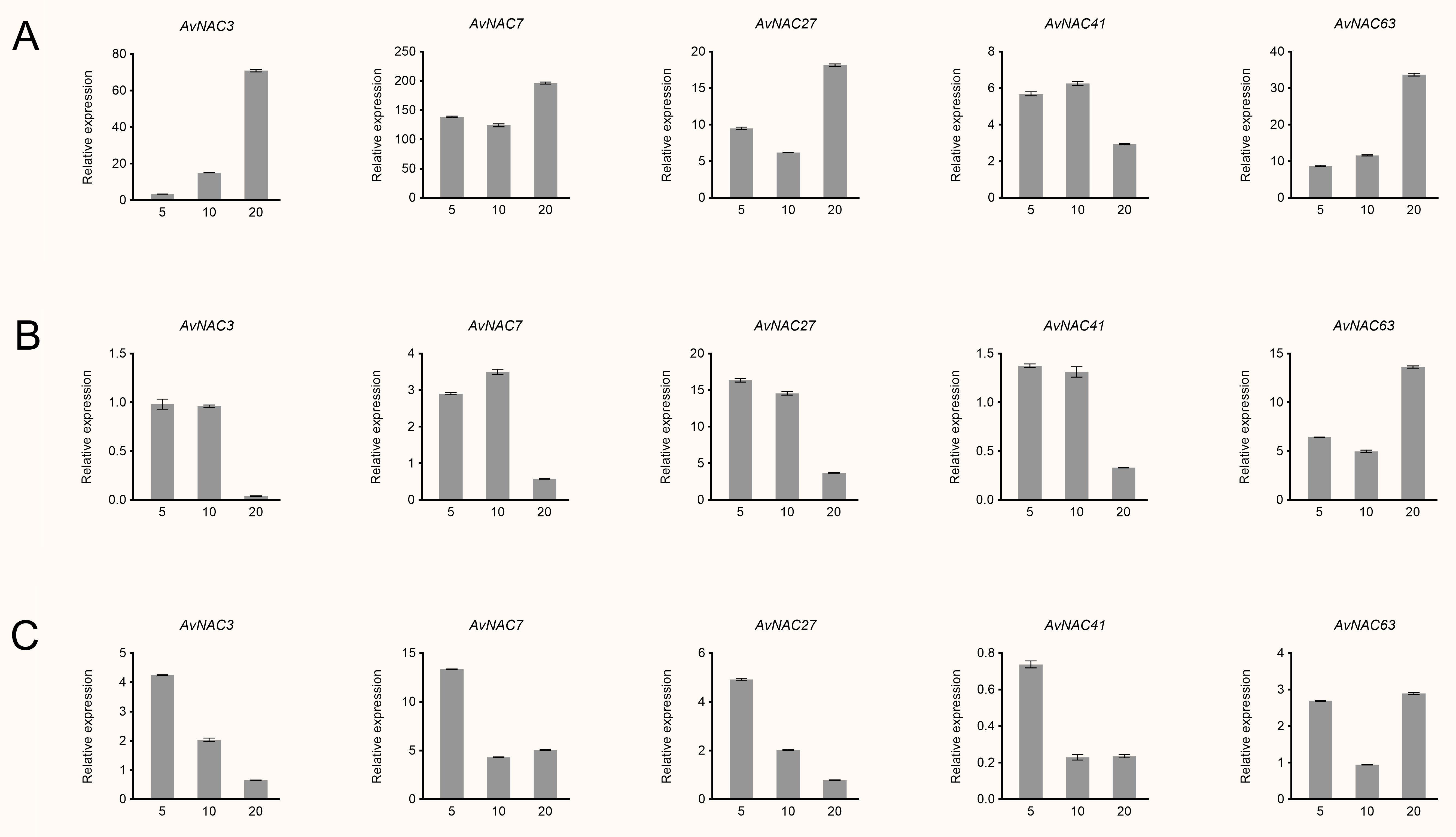

Supplement: Supplementary file 1 [file ijms-24-04578-s001.zip › Supplementary files/Supplementary Figure S1.tif]

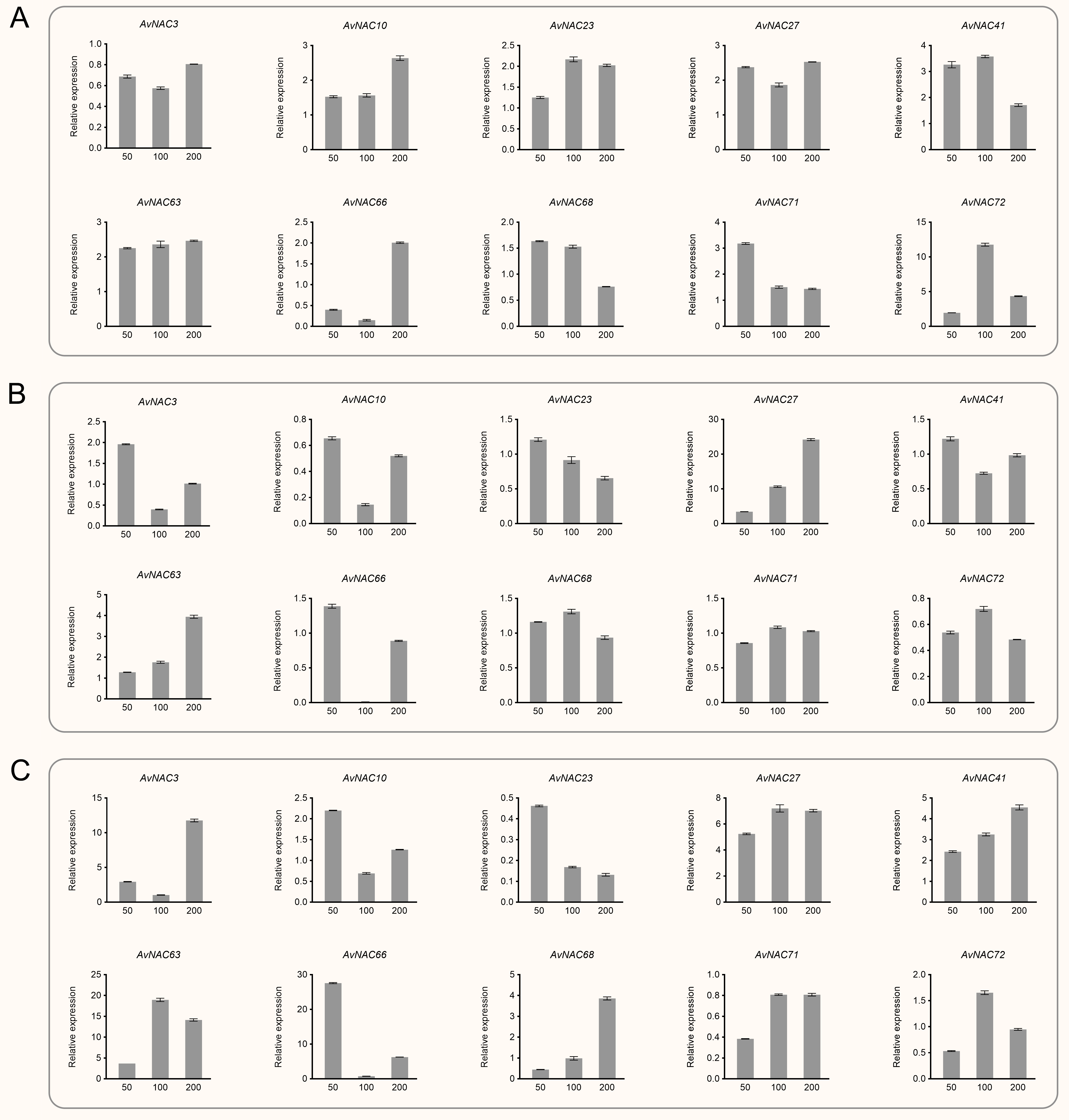

Supplement: Supplementary file 1 [file ijms-24-04578-s001.zip › Supplementary files/Supplementary Figure S2.tif]
